# The Impact of Unmet ADL Need on the Self-Rated Health and Life Satisfaction of Chinese Older Adults

**DOI:** 10.1093/geroni/igab046.3203

**Published:** 2021-12-17

**Authors:** Jing Huang, Edmond Pui Hang Choi, Pui Hing Chau

**Affiliations:** 1 School of Nursing, The University of Hong Kong; 2 The University of Hong Kong, Hong Kong, Hong Kong

## Abstract

This study aims to examine the associations of change in unmet need for assistance with Activities of Daily Living (ADL) with the self-rated health and life satisfaction of community-dwelling Chinese older adults. Using national longitudinal data from the Chinese Longitudinal Healthy Longevity Study, we examined the associations of unmet ADL need with self-rated health and with life satisfaction from baseline (T1) to a 3-year follow-up (T2) among 1,914 older adults with ADL limitation. Change in unmet ADL need was categorized into “Persistently Unmet”, “Unmet at T1 Only”, “Unmet at T2 Only”, and “Never Unmet”. Self-rated health and life satisfaction were rated by 5-point Likert scales. Linear mixed models were performed to examine the associations, controlling for sociodemographic factors, health conditions, and social support. The results showed that older adults whose ADL needs were persistently unmet, those unmet at T2 only, and those never unmet, experienced a significant decline in self-rated health from baseline to follow-up, but those unmet at T1 only experienced a significant rise in self-rated health. While the life satisfaction was stable from baseline to follow-up among older adults whose ADL needs were persistently unmet or never unmet, it significantly decreased among those unmet at T2 only and significantly increased among those unmet at T1 only. The effects of unmet ADL need on self-rated health and life satisfaction appeared to be short-term rather than long-term. These findings facilitate a better understanding of unmet ADL need and emphasize the importance to fully meet the ADL needs of older adults.

